# Yellow Fever at New Orleans

**Published:** 1905-09

**Authors:** 


A Monthly Review of Medicine and Surgery.

*EDITOR:*

WILLIAM WARREN POTTER, M. D.

All communications, whether of a literary or business nature, books for review and exchanges, should be addressed to the editor 284 Franklin St., Buffalo, N. Y.

Vol. Lxi. SEPTEMBER, 1905. No. 2

### Yellow Fever at New Orleans.

THE sudden outbreak of yellow fever at New Orleans in the latter days of July took the people of the country by surprise; for, since General Wood stamped out the disease at Havana during his military governorship of Cuba, we had begun to think ourselves free from danger of any future invasion. We had perhaps forgotten that there were other sources for breeding the disease and hence had become lethargic.

We should have remembered, however, that whenever yellow fever has invaded the United States, it has been through the Mississippi delta and generally the first cases are landed at the port of New Orleans. The great fruit trade between Central American cities and New Orleans and the liability of the ships of that traffic to bring with them the deadly mosquitoe,—the stegomyia fasciata,—which has been pronounced as the only carrier of the infection, should have kept the quarantine officers alive to the possibility of the approach of the malady through that channel.

Since the fact has been firmly established that infected mosquitoes of the species above mentioned are the only carriers of the virus, it would seem imperative that every ship coming from the tropics should be fumigated at the port of departure and at the port of arrival. Nor should this be done in a perfunctory manner. Inasmuch as the fumigation is designed to kill off the mosquitoes, it should be ascertained that all the insects are dead before the fumigation is pronounced sufficient to release the vessel from detention. All this, however, is as well known to the quarantine officers as to anybody, hence we need not further pursue this point.

We cannot but sympathise with New Orleans in its serious affliction as well as in its humiliation because of the lack of enforcement of proper quarantine regulations. The loss of life has been large and the sickness has been appalling, the death of Archbishop Chapelle affording the saddest climax to this melancholy scene. The state authorities finally appealed to the general government and the President instructed Surgeon-General Walter Wyman, of the Public Health and Marine Hospital Service, an expert of the highest type, to assume charge of the campaign against yellow fever in the infected district. General Wyman immediately put Dr. J. H. White, of the service, in charge, and he has been most actively engaged for the last three weeks or more in an attempt to control the spread of the scourge.

Singularly enough, even in the face of the tragedy being enacted, there is a comic side to the picture. One, Dr. R. B. Leach, hailing from Saint Paul, invited himself to New Orleans to stay the progress of the epidemic, by administering pilules of arsenic to prevent or cut short yellow fever in an infected individual. Singularly enough, too, Governor Blanchard, after having surrendered sanitary control to the general government, issued a permit to Leach. In this we think the governor committed an error of tactics, if not of courtesy. The upshot of the matter, however, is that Leach and his method is discredited, Surgeon-General Wyman refusing to appoint a commission to carry out the tests demanded by Dr. Leach. This should bring to an end this silly business.

At the present writing news from the crescent city indicates that the epidemic is under control and the further spread of the disease is not to be apprehended,—a consummation most devoutly to be wished. The deaths August 24, numbered seven and the new cases forty-four. Dr. Edmond Souchon, president of the state board of health and a sanitarian of high authority, is coöperating with the government officer in charge, and everything pertaining to the details of the campaign appears to be running smoothly. Dr. J. Guiteras, the yellow fever expert, predicts the daily decrease of cases from this out, and that by the middle of September only sporadic cases will remain.

---

The only drawback to Dr. Leach's system of preventing yellow fever, says *The Tribune,* which has yet been discovered, is that it doesn't prevent. Dr. White, who is in charge of the United States Marine Hospital Service in New Orleans, reports several cases of the disease among the persons who have taken arsenic tablets for the period that was expected to insure immunity.

